# Deciphering the genetic landscape of seedling drought stress tolerance in wheat (*Triticum aestivum* L.) through genome-wide association studies

**DOI:** 10.3389/fpls.2024.1351075

**Published:** 2024-03-04

**Authors:** Santosh Gudi, Priyanka Halladakeri, Gurjeet Singh, Pradeep Kumar, Satinder Singh, Khairiah Mubarak Alwutayd, Diaa Abd El-Moneim, Achla Sharma

**Affiliations:** ^1^ Department of Plant Breeding and Genetics, Punjab Agricultural University, Ludhiana, Punjab, India; ^2^ Department of Genetics and Plant Breeding, Anand Agricultural University, Anand, India; ^3^ Texas A&M University, AgriLife Research Center, Beaumont, TX, United States; ^4^ Department of Agronomy, Horticulture, and Plant Science, South Dakota State University, Brookings, SD, United States; ^5^ Department of Biology, College of Science, Princess Nourah bint Abdulrahman University, Riyadh, Saudi Arabia; ^6^ Department of Plant Production (Genetic Branch), Faculty of Environmental Agricultural Sciences, Arish University, El-Arish, Egypt

**Keywords:** wheat, drought stress, root length, coleoptile length, GWAS, candidate genes

## Abstract

Wheat is an important cereal crop constrained by several biotic and abiotic stresses including drought stress. Understating the effect of drought stress and the genetic basis of stress tolerance is important to develop drought resilient, high-yielding wheat cultivars. In this study, we investigated the effects of drought stress on seedling characteristics in an association panel consisting of 198 germplasm lines. Our findings revealed that drought stress had a detrimental effect on all the seedling characteristics under investigation with a maximum effect on shoot length (50.94% reduction) and the minimum effect on germination percentage (7.9% reduction). To gain a deeper understanding, we conducted a genome-wide association analysis using 12,511 single nucleotide polymorphisms (SNPs), which led to the identification of 39 marker-trait associations (MTAs). Of these 39 MTAs, 13 were particularly noteworthy as they accounted for >10% of the phenotypic variance with a LOD score >5. These high-confidence MTAs were further utilized to extract 216 candidate gene (CGs) models within 1 Mb regions. Gene annotation and functional characterization identified 83 CGs with functional relevance to drought stress. These genes encoded the WD40 repeat domain, Myb/SANT-like domain, WSD1-like domain, BTB/POZ domain, Protein kinase domain, Cytochrome P450, Leucine-rich repeat domain superfamily, BURP domain, Calmodulin-binding protein60, Ubiquitin-like domain, etc. Findings from this study hold significant promise for wheat breeders as they provide direct assistance in selecting lines harboring favorable alleles for improved drought stress tolerance. Additionally, the identified SNPs and CGs will enable marker-assisted selection of potential genomic regions associated with enhanced drought stress tolerance in wheat.

## 1 Introduction

Drought is an important abiotic stress which negatively affects crop growth and development. The impact and severity of drought stress depend on the crop developmental stage, the duration and intensity of the drought, and the genetic makeup of a cultivar (Tanin et al., 2022; Ramesh et al., 2024). Drought stress at early developmental stages affects the seed germination by limiting water absorption, alters the mobilization of stored reserves, and affects the synthesis of proteins in germinating embryos (Almansouri et al., 2001). Additionally, at this stage drought stress reduces the photosynthetic efficiency, which results in the poor plant establishment with stunted growth (Tomar and Kumar, 2004). In contrast, drought stress at the later developmental stages (viz., anthesis and grain filling stage) reduces the biomass accumulation and grain filling duration, which ultimately results in reduced grain weight and grain yield per unit area (Senapati et al., 2019).

Wheat (*Triticum aestivum* L.) is a globally important cereal crop that is predominantly grown in the arid and semi-arid regions of the world, where water scarcity poses a significant challenge to agricultural production (Tanin et al., 2022). Wheat production in these regions is further challenged by the frequent occurrence of drought episodes which is the results of changing climatic conditions coupled with depleting water tables (da Silva et al., 2019). For instance, the severity and spread of heat wave in northern and central India in 2022-23 cropping season, accompanied by a lack of precipitation during March and April, significantly reduced the wheat yield by 4.41% (Bal et al., 2022). During this period, the average rainfall was found to be 60-99% lower than usual. Similarly, a frequent occurrence of drought episodes in the United States and Europe is threatening the winter wheat production (Toreti et al., 2023). For instance, incidence of drought stress at various developmental stages reduce the global wheat production by 35% (Gupta et al., 2020). Similarly, the evaluation of advanced breeding lines grown under drought stress showed a reduction in grain yield up to 45% as compared to controlled conditions. Therefore, to overcome these challenges and to feed the ever-rising human population, it is utmost important to prioritize the research objectives to develop drought-resilient wheat varieties (Bakala et al., 2020). However, breeding drought-resilient cultivars is challenged by the complex genetic architecture of drought stress tolerance. Nevertheless, the advanced molecular breeding tools such as quantitative trait loci (QTL) mapping, genome-wide association studies (GWAS), and genomic selection (GS) overcome these barriers and help in improving the quantitative traits (Gudi et al., 2022a; Zhang et al., 2022; Singh et al., 2022a, Singh et al., 2022b; Song et al., 2023).

Genome-wide association studies (GWAS) have greatly advanced our understanding of the genetic basis of complex traits. GWAS relies on genome-wide markers and exploits the historical recombinant events to identify significant marker-trait associations (MTAs) associated with targeted traits. GWAS has been successfully employed in identifying significant MTAs associated with grain yield (Gill et al., 2022), chlorophyll fluorescence (Gudi et al., 2023), disease resistance (Arora et al., 2019), and stress tolerance (Gudi et al., 2023) traits in wheat. GWAS has also been employed to identify the candidate genomic regions associated with drought stress tolerance in wheat. For instance, the GWAS was used to identify and clone a transcriptional factor, *TaWD40-4B.1*, responsible for increased grain yield under drought stress in wheat (Tanin et al., 2022). In addition, GWAS was used to identify the candidate genes (CGs) responsible for abscisic acid accumulation (Kalladan et al., 2017), proline accumulation (Verslues et al., 2014), root traits (Fatima et al., 2021), and biomass accumulation (Fatima et al., 2021) under drought stress.

Most of these studies focused on terminal drought stress, with limited studies involving seedling drought stress tolerance. Since wheat seedlings are crucial in determining plant stand and grain yield, researchers are now prioritizing their studies at seedling stage. For instance, multiple GWAS studies identified the significant MTAs associated with germination percentage (GP), shoot length (SL) (Maulana et al., 2020), root length (RL) (Ayalew et al., 2017), coleoptile length (CL) (Ma et al., 2020), seedling vigour (SV) (Maulana et al., 2021), and drought tolerance index (DTI) (Schierenbeck et al., 2023) under drought stress. These studies identified the candidate genomic regions associated with seedling drought stress tolerance. Despite these achievements, further research is required to fully understand the genetic and molecular mechanisms involving seedling drought stress tolerance in wheat.

In order to achieve this goal, we investigated the effect of drought stress on a collection of 198 wheat germplasm lines at the seedling stage. We also carried genome-wide association studies (GWAS) to identify the candidate genomic regions associated with seedling traits under drought stress. Additionally, we carried candidate gene (CG) analysis to identify the potential CGs associated with seedling drought stress tolerance.

## 2 Materials and methods

### 2.1 Plant material and experimental design

The wheat association panel used in this study involves 198 genotypes, including released varieties (from 1905 to 2020), landraces, and advanced breeding lines (**Supplementary Table 1**). Germplasm lines were evaluated for seedling drought stress tolerance using 20% polyethylene glycol 6000 (PEG-6000) in completely randomized design (CRD) with three replications. Seedling drought stress tolerance was assessed using the modified Cigar roll method of seed germination (Sharma et al., 2022). In brief, 20 uniform and bold seeds from each genotype were surface sterilized with 0.1% HgCl_2_ for 30 minutes and subsequently washed three times in distilled water. Seeds were then placed on germination paper moistened with distilled water (for control treatment) and 20% PEG-6000 (for drought treatment; to ensure drought stress from germination itself). Germination papers were rolled and kept vertically in dark chambers for 2-3 days until germination. Following the seed germination, the growth chambers were adjusted to a photoperiod of 16-hour of light and 8-hour of darkness with 25˚C day/night temperature for 12 days. Data on the germination percentage (GP; %), shoot length (SL; cm), root length (RL; cm), and coleoptile length (CL; cm) were recorded on the 12^th^ day after germination. Seedling vigour (SV) was calculated by using the following formula:


$$\begin{array}{c} \text{Seedling vigour }\left(\text{SV}\right)=\;\\ \frac{\left[\text{shoot length }\left(\text{cm}\right)+\text{root length }\left(\text{cm}\right)\right]\;\times \text{germination percentage }\left(\%\right)}{100} \end{array}$$

### 2.2 Phenotypic data analysis

Phenotypic data on seedling traits were subjected mixed linear model (MLM) using “META-R version 6.04” to obtain the best linear unbiased estimations (BLUEs) (Alvarado et al., 2020). BLUE values were calculated by using the following formula:


Yij=µ+Gi+Rj+eij

Where, Yij: seedling trait; µ: grand mean; Gi: effect of i^th^ genotype; Rj: effect of j^th^ replication; eij: error associated with i^th^ genotype and j^th^ replication, which is assumed to be normally and independently distributed, with mean zero and homogeneity of variances as σ^2^.

The following formula was used to obtain the broad-sense heritability (h^2^_bs_) for seedling traits in META-R:


$${\text{h}}_{\text{bs}}^{2}=\frac{{\text{σ}}_{\text{g}}^{2}}{{\text{σ}}_{\text{g}}^{2}+{\text{σ}}_{\text{e}}^{2}/R}$$

Where, h _bs_^2^: broad-sense heritability; σ_g_^2^: genetic variance; σ_g_^2^: error variance; and R: number of replications.

Pearson’s correlation coefficient analysis and principal component analysis (PCA) were carried, respectively, using the “Corrplot” and “Factoextra” package built in the RStudio (Team, 2016).

### 2.3 Single nucleotide polymorphism genotyping and population structure analysis

The standard CTAB (Cetyl trimethyl ammonium bromide) method was employed to extract high-quality genomic DNA from the young leaves of all the germplasm lines using the “2010 Geno/Grinder®”. DNA quantification was done using a NanoDrop™ spectrophotometer, whereas the quality was assessed using 0.8 percent agarose gel electrophoresis. Single nucleotide polymorphism (SNP) genotyping of association panel (i.e., 198 genotypes) was outsourced from a private firm (from the Imperial life sciences, India) using a commercial Wheat Breeder’s Genotyping Array which is also referred as Affymetrix Axiom^®^ genotyping array. It is a high-density genotyping platform featuring 35,143 SNPs distributed across the wheat genome (Allen et al., 2017). Subsequently, the genotypic data was filtered to remove the: (i) monomorphic SNPs; (ii) SNPs with minor allele frequency (MAF) of less than 5%; (iii) SNPs with more than 20% missing data; and (iv) SNPs with heterozygote frequency greater than 20% (Gudi et al., 2023).

Model-based clustering, principal component analysis (PCA), and kinship analysis was used for population structure analysis. In brief, the Bayesian approach built in the “STRUCTURE v.2.3.4” was used to assess the number of sub-groups in the panel with a K-value set to 10 (Pritchard et al., 2000). Each K-value was investigated for three times with 100,000 burn-in iterations and 100,000 Markov Chain Monte Carlo (MCMC) replications. The optimal number of subpopulations was determined using *ad-hoc* and Evanno’s methods in the “STRUCTURE HARVESTER v0.6.9450” software (Evanno et al., 2005). SNP based PCA was carried out using “TASSEL v5.2.8”, and a 3D plot was generated using RStudio (Bradbury et al., 2007). Kinship analysis was done in the “GAPIT software”, and the resulting square matrix was used to build a kinship plot (Lipka et al., 2012).

### 2.4 Genome-wide association study, linkage disequilibrium, and candidate genes analysis

The BLUE values along with genotypic data were used for GWAS analysis using “mrMLM v4.0.2.” software (Zhang et al., 2020). It is a multi-locus GWAS platform integrated with six multi-locus models (viz., mrMLM, FASTmrMLM, FASTmrEMMA, pLARmEB, pKWmEB, and ISIS EM-BLASSO). mrMLM relies on multiple algorithms to select all the possible markers associated with trait of interest. Once the potential markers are selected, they will be put in multi-locus genetic model to estimate all the effects by empirical Bayes. Further, all the non-zero effects are identified by likelihood ratio test for true MTAs and those MTAs identified in at least two models will be considered as significant MTAs (highlighted with pink color in the Manhattan plot). Though a less stringent criteria is adopted for identifying MTAs, mrMLM have high power and accuracy, with a low false positive rate. Furthermore, the MTAs which had the LOD scores >5 and explained >10% phenotypic variation were considered as high-confidence MTAs. Finally, the chromosome wise linkage disequilibrium (LD) analysis was carried out from the 100 Kb region, and the LD decay plots were generated using RStudio.

The candidate genes (CGs) models were extracted from the high-confidence MTAs which explained more than 10% of the phenotypic variation and had the LOD scores >5 using the “BioMart” tool present in the “Ensembl Plants database”. The 1Mb genomic region (total 2 Mb region) on either side of the SNPs from the IWGSC Chinese Spring RefSeq v1.1 was used to extract the CG models. The “InterPro database” was used to obtain functional descriptions of the identified gene models.

## 3 Results

### 3.1 Assessing the effect of drought stress on seedling characteristics

**Figure 1:**
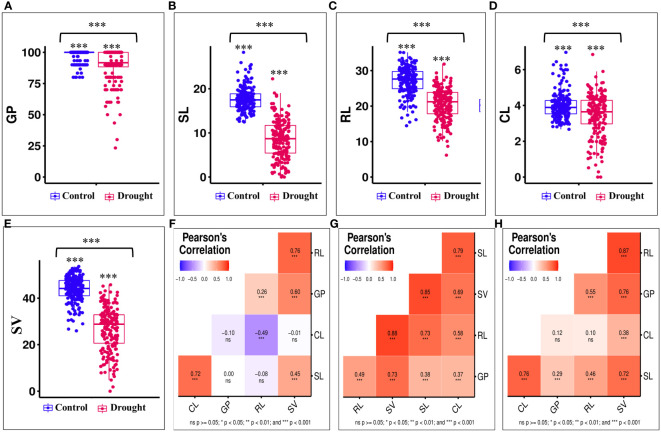
Effect of drought stress on seedling characteristics and Pearson’s correlation coefficient analysis: **(A–E)** effect of drought stress on germination percentage (GP, %), shoot length (SL, cm), root length (RL, cm), coleoptile length (CL, cm), and seedling vigour (SV); **(F–H)** Pearson’s correlation coefficient analysis for seedling characteristics in control; drought; and overall mean. Level of significance obtained through Fisher's LSD test (0.001 ‘***’; 0.01 ‘**’; 0.05 ‘*’).

Association panel was evaluated for seedling traits and the data on seedling characteristics, including GP, SL, RL, and CL were collected from the controlled and drought stress conditions. Seedling vigor (SV) was calculated using the aforementioned formula. We observed a significant variation (P-value<0.01) for all the seedling traits under controlled and drought stress condition (**Figure 1**). It was also noticed that the genotypes showed higher variation under drought stress than under controlled condition. This suggests the significant effect of drought stress on seedling characteristics. The values of the investigated traits under controlled conditions and drought stress ranged respectively as follow: 70-100% and 10-100% for GP; 12.1-28.74 and 0-23.9 cm for SL; 13.75-35.64 and 5.55-32.8 cm for RL; 2.65-6.97 and 0-6.93 cm for CL, and 21.85-54.03 and 1.11-46.6 SV (**Figures 1A–E**). The GP showed the lowest coefficient of variance (CV) under both control (4.59) and drought stress (15.14), whereas the CV was the highest for CL (18.95) and SL (49.9) under control and drought stress, respectively (**Table 1**). Furthermore, the GP exhibited the lowest heritability (broad sense) under control and drought stress conditions. In contrast, the CL displayed the highest heritability under control condition, while the SL showed the highest heritability under drought stress (**Table 1**). The principal component analysis (PCA) revealed that the first two PCs explained 81.38 and 88.01 percent of the total phenotypic variance, respectively, under control and drought stress condition (**Supplementary Table 2**; **Supplementary Figures 1A–D**).

**Table 1:** Descriptive statistical analysis for seedling characteristics under control and drought stress condition.

Traits	Mean	Range	LSD at 5%	CV	Heritability (bs)
Control					
GP	97.44	70-100	6.53	4.59	0.55
SL	17.75	12.1-28.74	0.69	14.78	0.97
RL	27.18	13.75-35.64	0.8	13.57	0.98
CL	4	2.65-6.97	0.12	18.95	0.99
SV	43.83	21.85-54.03	3.14	11.55	0.87
Drought (20% PEG)					
GP	89.75	10-100	10.22	15.14	0.82
SL	8.71	0-23.9	0.96	49.9	0.98
RL	20.66	5.55-32.8	1.16	20.62	0.97
CL	3.49	0-6.93	0.29	31.39	0.97
SV	26.88	1.11-46.6	3.19	33.69	0.95

LSD, least significant difference; CV, coefficient of variance; bs, broad sense; PEG, polyethylene glycol; GP, germination percentage; SL, shoot length; RL, root length; CL, coleoptile length; and SV, seedling vigour.

Drought stress significantly reduced all the traits with a maximum effect on SL (50.93%) and the minimum effect on GP (7.89%). However, the effect of drought stress was intermediate on other traits, with a reduction of 23.98% for RL, 12.75% for CL, and 38.67% for SV. Pearson’s correlation coefficient analysis revealed the significant positive association (P-value<0.05) between studied traits (**Figures 1F–H**). Interestingly, the level of correlation was found to be higher under drought stress than under controlled condition. For instance, significant positive correlation was observed for: SV with SL (r = 0.45), GP (r = 0.6), and RL (r = 0.76); RL with GP (r = 0.26); and SL with CL (r = 0.72) under controlled condition (**Figure 1F**). Furthermore, we found a significant negative correlation (r = -0.49) between RL and CL. Similarly, we observed the significant positive association for all the traits under drought stress with maximum association between SV and RL (r = 0.88) and minimum association between GP and CL (r = 0.37) (**Figure 1G**). The high level of correlation between the studied traits was further supported by the PCA biplots (**Supplementary Figure 1**).

### 3.2 SNP genotyping and population structure analysis

**Figure 2:**
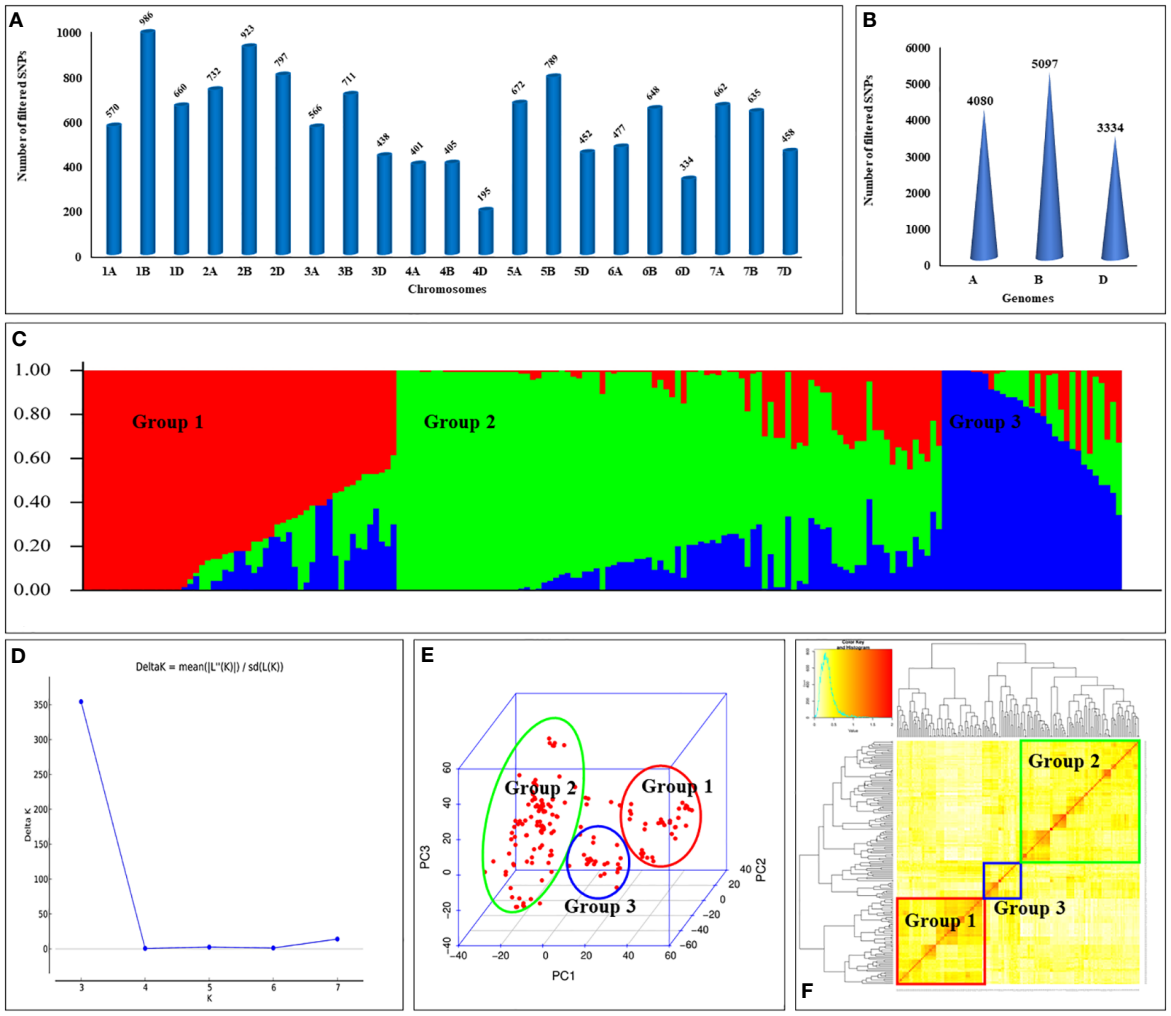
Genomic features of wheat association panel: **(A)** number of SNPs per chromosome; **(B)** number of SNPs in A, B, and D sub-genomes; **(C)** sub-populations present in the association panel; **(D)** DeltaK plot showing number of sub-populations; **(E)** principal component analysis (PCA); and **(F)** kinship analysis for the association panel.

Association panel was genotyped using the Wheat Breeder’s Genotyping Array to get the genotypic information on a total of 35,143 SNPs. Raw data was subjected to filtering to remove the SNPs with minor allele frequency (MAF) less than 5% (12,569 SNPs), heterozygosity exceeding 20% (10,044 SNPs), missing data exceeding 20% (9 SNPs), and unknown chromosomal information (1,010). This resulted in a final set of 12,511 high-quality SNPs which was used for GWAS. Association panel showed the variation in SNP distribution across the chromosomes and the sub-genomes. For instance, chromosome 1B displayed the highest number of SNPs (986), whereas chromosome 4D had the lowest number (195) (**Figure 2A**). Similarly, B-genome displayed the highest number of SNPs (5,097), while the D-genome displayed the lowest number of SNPs (3,334) (**Figure 2B**).

Population structure analysis revealed the presence of three distinct subpopulations (i.e., K=3) in the panel (**Figures 2C–F**). Notably, subpopulation II displayed the highest number of genotypes, comprising a total of 94 individuals, while subpopulation III had the fewest genotypes, with only 31 individuals. These findings were further confirmed by the principal component analysis (PCA) (**Figure 2E**) and kinship analysis (**Figure 2F**).

### 3.3 Genome-wide association studies and linkage disequilibrium analysis

**Figure 3:**
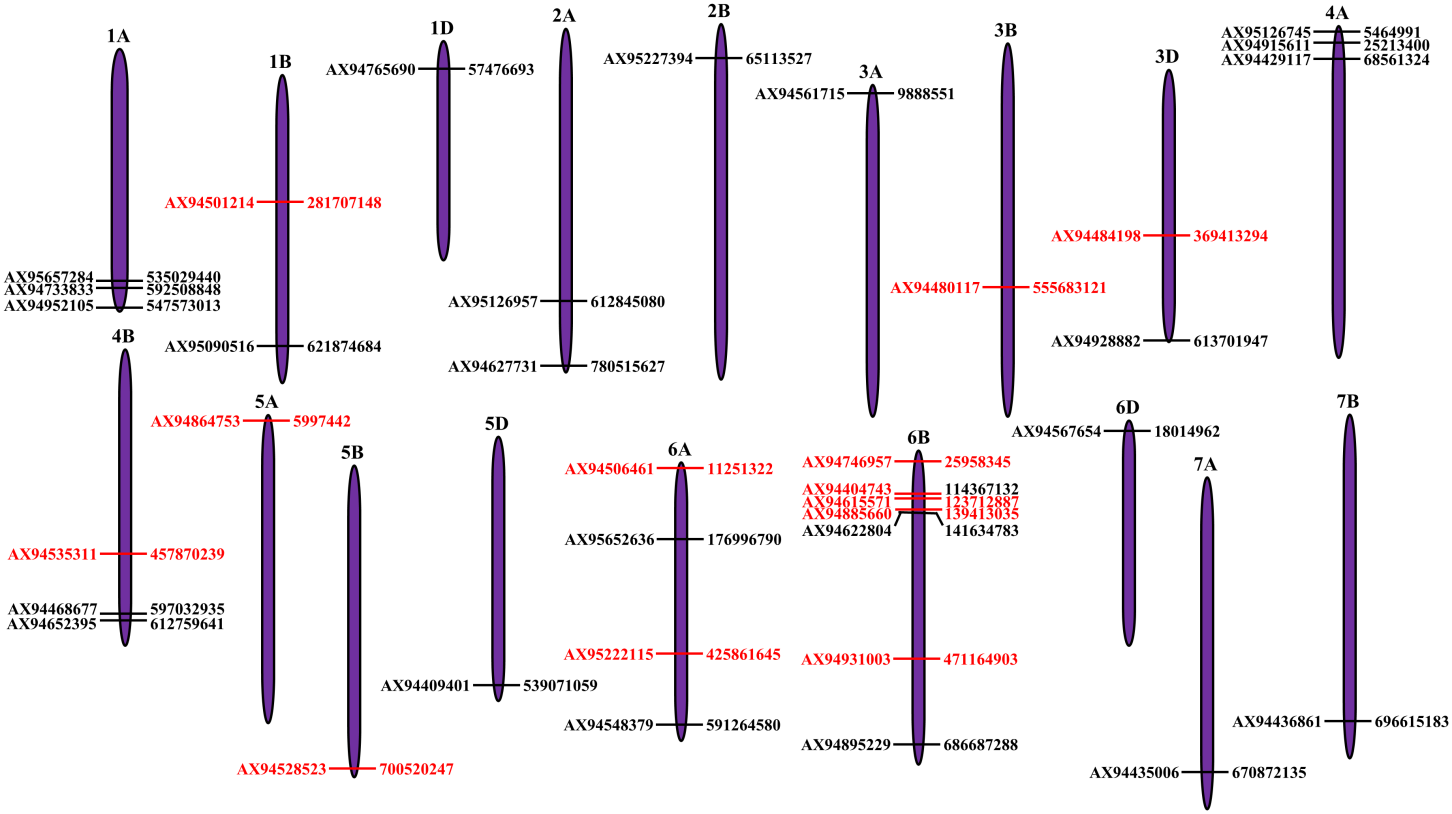
Distribution of quantitative trait loci (QTLs) identified for seedling traits under control and drought stress condition on the wheat chromosomes. Each MTA is depicted as horizontal bar with SNP name at the left side and its physical position (bp) at right side. The MTAs highlighted in red color represent high-confidence MTAs which explained the phenotypic variation of more than 10% and had the LOD score of more than five.

Genome-wide association studies (GWAS) identified 39 MTAs associated with all seedling traits. out of which, 18 MTAs were identified under controlled condition, whereas 21 MTAs were identified under drought stress (**Figure 3**; **Supplementary Table 3**). Of the 18 MTAs identified under controlled conditions, two MTAs were associated with GP (**Supplementary Figure 2A**), five MTAs were associated with SL (**Supplementary Figure 3A**), RL (**Supplementary Figure 4A**), and CL (**Supplementary Figure 5A**), and one MTA was associated with SV (**Supplementary Figure 6A**). Similarly, of the 21 MTAs identified under drought stress, five MTAs were associated with GP (**Supplementary Figure 2B**), three MTAs were associated with SL (**Supplementary Figure 3B**) and RL (**Supplementary Figure 4B**), four MTAs were associated with CL (**Supplementary Figure 5B**), and six MTAs were associated with SV (**Supplementary Figure 6B**). Three of these 39 MTAs were associated with multiple traits and hence they were pleiotropic in nature. For instance: (i) MTA located on chromosome 4A (viz., AX95126745) was associated with GP and SV; (ii) MTA located on chromosome 5A (AX94864753) was associated with SL and SV; and (iii) MTA located on chromosome 6A (AX95222115) was associated with RL and CL. Among all, only 19 MTAs explained more than 10% of the phenotypic variation and they were considered as major QTLs. Out of the 19 major QTL, 12 showed a LOD score >5, which were considered as high-confidence MTAs (**Table 2**; **Figure 3**).

**Table 2:** List of high-confidence quantitative trait loci (QTLs) identified for seedling characteristics under control and drought stress conditions.

QTLs/SNPs	Trait	Treatment	Chromosome	SNP position (bp)	LOD score	PVE (%)
AX94484198	Germination percentage (GP)	Drought	3D	369413294	6.16	23.83
AX94615571			6B	123712887	8.98	16.13
AX95222115	Root length (RL)	Control	6A	425861645	6.13	12.15
AX94404743			6B	114367132	7.79	17.59
AX94501214		Drought	1B	281707148	5.75	31.73
AX94480117	Shoot length (SL)	Control	3B	555683121	6.28	15.66
AX94864753			5A	5997442	5.73	11.61
AX94931003			6B	471164903	5.6	31.19
AX94506461		Drought	6A	11251322	7.01	13.74
AX94535311	Coleoptile length (CL)	Control	4B	457870239	12.79	22.35
AX94528523			5B	700520247	5.8	11.81
AX95222115			6A	425861645	10.86	15.36
AX94885660		Drought	6B	139413035	6.77	36.91

QTLs, quantitative trait loci; SNPs, single nucleotide polymorphism; bp, base pair; LOD, log of odds; PVE, phenotypic variance explained.

Linkage disequilibrium (LD) analysis revealed a substantial variation in LD decay across the chromosomes and the sub-genomes (**Supplementary Figure 7**). For instance, chromosome-level LD analysis revealed the largest LD block size in chromosome 1D (35.07 Mb) and the smallest LD block size in chromosome 7D (1.12 Mb). On average, each chromosome displayed an LD block size of 8.71 Mb. Similarly, LD analysis revealed the largest LD block in the B-genome (11.88 Mb) and the smallest LD block in the A-genome (5.91 Mb). However, at the whole genome level we observed the an average LD block size of 8.43 Mb (**Supplementary Figure 7**).

### 3.4 Candidate gene analysis

A total of 12 high-confidence MTAs explaining >10% of the phenotypic variance and with a LOD score >5 were utilized to extract 216 unique gene models (**Supplementary Table 4**). The highest number of gene models (36 genes) was found on the MTA located on chromosome 5B (viz., AX94528523). Conversely, the lowest number of gene models (only 2 genes) were identified on the MTA located on chromosome 1B (viz., AX94501214). Gene annotation and functional characterization identified 83 gene models with functional relevance to drought stress tolerance and were considered as the putative candidate genes (CGs) (**Table 3**). These putative CGs encodes for a diverse range of proteins, including, WD40 repeat domain, Myb/SANT-like domain, WSD1-like domain, BTB/POZ domain, protein kinase domain, cytochrome P450, leucine-rich repeat domain superfamily, BURP domain, calmodulin-binding protein60, ubiquitin-like domain, etc.

**Table 3:** List of potential candidate genes (CGs) identified from the high-confidence quantitative trait loci (QTLs) for seedling traits under drought stress condition.

QTLs/SNPs	Gene ID	Gene position (bp)	Interpro Description
AX94480117	TraesCS3B02G345300	555148343-555149367	Protein kinase domain
	TraesCS3B02G345700	555270080-555273699	WD40 repeat
	TraesCS3B02G345400	555155004-555156101	BTB/POZ domain
	TraesCS3B02G346800	556367760-556369658	F-box domain
	TraesCS3B02G345600	555243898-555244989	BTB/POZ domain
	TraesCS3B02G347000	556487647-556519303	Histidine phosphatase superfamily, clade-2
	TraesCS3B02G346700	556349511-556351253	Cytochrome P450
AX94484198	TraesCS3D02G266700	370108377-370112784	Sde2, N-terminal ubiquitin domain
	TraesCS3D02G266900	370123379-370126365	O-acyltransferase, WSD1-like, N-terminal
AX94535311	TraesCS4B02G216900	457049370-457052911	Phosphatidylinositol-4-phosphate 5-kinase, core
	TraesCS4B02G217300	458124485-458126550	Phospholipase A2
	TraesCS4B02G217400	458456678-458460338	Myc-type, basic helix-loop-helix (bHLH) domain
AX94864753	TraesCS5A02G008700	6287359-6290472	Cytochrome P450
	TraesCS5A02G008800	6411078-6413189	Cytochrome P450
	TraesCS5A02G008900	6422019-6424272	Cytochrome P450
	TraesCS5A02G009000	6438667-6440111	Tetratricopeptide repeat 1
	TraesCS5A02G009100	6444931-6449572	Ankyrin repeat
	TraesCS5A02G009200	6482289-6488020	Ankyrin repeat
	TraesCS5A02G009700	6614883-6620347	Ankyrin repeat
	TraesCS5A02G009800	6621498-6628092	Ankyrin repeat
	TraesCS5A02G009900	6663221-6663596	Calreticulin/calnexin
	TraesCS5A02G010000	6665804-6668336	Zinc finger, RING-type
	TraesCS5A02G010400	6773230-6774222	Transcription factor, MADS-box
	TraesCS5A02G010500	6797997-6799208	ELO family
	TraesCS5A02G010600	6982513-6983531	Casparian strip membrane protein domain
AX94528523	TraesCS5B02G550100	701519688-701520317	Protein kinase domain
	TraesCS5B02G548400	700945256-700947048	Cytochrome P450
	TraesCS5B02G546500	699720646-699725714	Phosphatidic acid phosphatase type 2/haloperoxidase
	TraesCS5B02G548600	701001929-701002519	Cytochrome P450
	TraesCS5B02G546700	699946003-699949487	3-beta hydroxysteroid dehydrogenase/isomerase
	TraesCS5B02G548100	700854870-700856601	Cytochrome P450
	TraesCS5B02G547800	700574102-700575217	Sterile alpha motif domain
	TraesCS5B02G548300	700936413-700939062	Cytochrome P450
	TraesCS5B02G548200	700917011-700918440	Zinc finger, RING-type
	TraesCS5B02G548700	701050114-701051876	Cytochrome P450
	TraesCS5B02G547200	700230739-700234958	NAD-dependent epimerase/dehydratase
	TraesCS5B02G549900	701465822-701466980	Myb/SANT-like domain
	TraesCS5B02G548500	700997146-700998633	Expansin
	TraesCS5B02G549000	701158488-701163796	Leucine-rich repeat domain superfamily
	TraesCS5B02G550000	701511918-701516803	Protein kinase domain
	TraesCS5B02G547300	700235845-700237592	F-box-like domain superfamily
	TraesCS5B02G546600	699729456-699731225	F-box domain
	TraesCS5B02G547000	700216666-700225371	3-beta hydroxysteroid dehydrogenase/isomerase
AX95222115	TraesCS6A02G226100	425741848-425746917	Zinc finger, ZZ-type
AX94506461	TraesCS6A02G021300	10491634-10496460	Putative S-adenosyl-L-methionine-dependent methyltransferase
	TraesCS6A02G021900	11006033-11007811	Leucine-rich repeat domain superfamily
	TraesCS6A02G022200	11157368-11159968	Cytochrome P450
	TraesCS6A02G022400	11231875-11236237	Protein kinase domain
	TraesCS6A02G022700	11280128-11280811	Transcription factor, MADS-box
	TraesCS6A02G023000	11496482-11500875	Leucine-rich repeat domain superfamily
	TraesCS6A02G023400	11679111-11681611	Leucine-rich repeat domain superfamily
	TraesCS6A02G023700	11790646-11791549	Zinc finger, RING-type
	TraesCS6A02G023800	11840405-11841583	F-box-like domain superfamily
	TraesCS6A02G023900	11883792-11886291	F-box-like domain superfamily
	TraesCS6A02G024000	11963972-11966414	Peptidase C78, ubiquitin fold modifier-specific peptidase 1/ 2
	TraesCS6A02G024100	12055217-12058516	Leucine-rich repeat
	TraesCS6A02G024200	12077818-12079854	Leucine-rich repeat
	TraesCS6A02G024300	12117881-12120124	Protein kinase domain
	TraesCS6A02G024400	12131874-12133343	Very-long-chain 3-ketoacyl-CoA synthase
AX94615571	TraesCS6B02G127600	123499909-123503951	Casparian strip membrane protein domain
	TraesCS6B02G127800	123702609-123715378	Protein kinase domain
	TraesCS6B02G128100	123747342-123751102	Protein kinase domain
	TraesCS6B02G128200	123829736-123836519	Diacylglycerol acyltransferase
	TraesCS6B02G128400	123910941-123913009	F-box domain
	TraesCS6B02G128500	124083997-124087349	F-box domain
	TraesCS6B02G128600	124091073-124091696	BURP domain
	TraesCS6B02G128700	124384674-124388848	BURP domain
AX94404743	TraesCS6B02G118800	113588038-113590079	Cytochrome P450
	TraesCS6B02G119700	114334445-114335913	F-box domain
	TraesCS6B02G119800	114366990-114372653	UBA-like superfamily
	TraesCS6B02G119900	114507380-114511418	CALMODULIN-BINDING PROTEIN60
	TraesCS6B02G120000	114879592-114880826	F-box-like domain superfamily
AX94931003	TraesCS6B02G261500	471232702-471235523	Thioredoxin domain
	TraesCS6B02G260300	470808922-470810315	F-box domain
	TraesCS6B02G260500	470825718-470831560	Pyridoxamine 5'-phosphate oxidase
	TraesCS6B02G261700	471306774-471314023	Peptidase S10, serine carboxypeptidase
	TraesCS6B02G261400	471180551-471181877	Tetratricopeptide-like helical domain superfamily
	TraesCS6B02G260100	470786021-470795563	Inositol monophosphatase-like
AX94885660	TraesCS6B02G140200	138643641-138645626	Cathepsin propeptide inhibitor domain (I29)
	TraesCS6B02G140400	139444367-139445136	Myb/SANT-like domain
	TraesCS6B02G140500	139883785-139886327	F-box domain
	TraesCS6B02G140600	139893280-139894535	Ubiquitin-like domain
	TraesCS6B02G140800	140331072-140333562	F-box domain

QTLs: quantitative trait loci; SNPs: single nucleotide polymorphism; bp: base pair.

## 4 Discussion

Drought is the major abiotic stress affecting the potential wheat production at various developmental stages. In the early developmental stages, drought stress affects the seed germination and seedling establishment by preventing the water and nutrient absorption (Almansouri et al., 2001; Tomar and Kumar, 2004). Drought stress also reduces the photosynthetic efficiency, leading to reduced biomass accumulation which ultimately leads to reduced grain yield per unit area. Plants have developed various mechanisms to alleviate the effects of drought stress (Qaseem et al., 2019). Understanding these mechanisms of drought stress tolerance and developing drought-resilient cultivars helps in reducing the yield losses. However, the quantitative nature and the complex genetic architecture of drought stress tolerance hinders the genetic improvement. With the advancements in novel genomics and molecular biology tools coupled with low-cost, high-throughput genome sequencing, it is now possible to identify and introgress the candidate genomic regions associated with drought stress tolerance. For instance, GWAS utilize the whole genome markers to identify the significant MTAs associated with drought tolerance in wheat. Once such MTAs are identified, they can be employed in marker-assisted breeding to transfer the candidate genomic regions in the background of commercial cultivars to develop drought-resilient cultivars (Gudi et al., 2020; Varshney et al., 2021). In order to achieve this, we assessed the impact of drought stress on various seedling traits in a wheat germplasm set comprising 198 lines. We also carried the GWAS and CG analysis to unravel the genetic mechanisms underlying seedling drought stress tolerance in wheat.

### 4.1 Effects of drought stress on seedling characteristics

Genetic variation is essential for crop improvement, as it provides breeders with diverse options to develop new crop varieties with improved traits and adaptability to changing climatic conditions (Singh et al., 2022a; Singh et al., 2022b; Singh et al., 2024). We observed a substantial amount of genetic variation in the association panel for the studied traits under controlled and drought stress condition (**Figure 1**). Multiple studies reported the larger genetic variation under drought stress than in normal condition (Ayalew et al., 2017; Ahmed et al., 2022; Singh et al., 2022c; Schierenbeck et al., 2023). Similarly, we observed the greater genetic variation for all the traits under drought stress (viz., GP: 10-100%; SL: 0-23.9 cm; RL: 5.55-32.8 cm; CL: 0-6.93 cm, and SV: 1.11-46.6) than the controlled condition (viz., GP: 70-100%; SL: 12.1-28.74 cm; RL: 13.75-35.64 cm; CL: 2.65-6.97 cm, and SV: 21.85-54.03). These findings highlight the significant impact of drought stress on seed germination and seedling establishment (**Figures 1A–E**). Previous studies have reported the detrimental effects of drought stress on various seedling traits. For instance, drought stress has been shown to reduce germination percentage by 12%, plumule length by 79%, radicle length by 59%, SL by 36%, RL by 11%, and seedling length by 36% (Mickky and Aldesuquy, 2017; Ahmed et al., 2022; Mahpara et al., 2022; Schierenbeck et al., 2023). In alignment with these findings, our study revealed a maximum effect of drought stress on SL (with 50.94% reduction) and the minimum effect on GP (with 7.9% reduction) which suggest the varying degree of sensitivity among different seedling traits to drought stress. Higher heritability values of quantitative traits aids plant breeders in selecting traits, predicting offspring performance, prioritizing breeding efforts, and improving crop varieties for enhanced drought tolerance in an efficient and targeted manner (Bernardo, 2020). Studies reported high level of heritability for the seedling traits under drought stress. For instance, Schierenbeck and co-authors (2023) reported high level of heritability for CL (98 and 97% respectively, under control and drought stress), SL (97 and 94% respectively, under control and drought stress), and RL (89 and 87% respectively, under control and drought stress). Similarly, we found the high heritability and correlation (among the traits) under controlled and drought stress condition for all the seedling traits (**Table 1**). These findings suggests that a substantial proportion of the observed phenotypic variation is under genetic control, which can be exploited to breed the drought-tolerant wheat varieties.

### 4.2 Population structure analysis

Population structure is the differences in allele frequency between subpopulations, which results from evolutionary forces that disturb Hardy-Weinberg equilibrium. Population structure introduces the confounding effects in association studies which leads to increased false positive associations. Therefore, it is crucial to include structure analysis to effectively circumvent or discern these false positive associations. Bayesian approaches and model-free methods, such as principal component analysis (PCA), have proven to be valuable tools in achieving this goal (Alotaibi et al., 2022). In the present study we applied both Bayesian approaches and PCA to investigate population structure existing in the association panel which revealed the presence of three distinct subpopulations (**Figure 2**). These subpopulations likely occurred due to the inclusion of exotic lines, Indian land races, and Indian released varieties or advanced breeding lines. We also observed the admixture within each subpopulation, indicating the genetic contribution from two or more ancestral parents. By including diverse germplasm lines in the association panel, we ensured sufficient allelic diversity, thus increasing the likelihood of detecting all possible allelic variants associated with targeted traits. Kinship analysis examines the familial relatedness among the germplasm lines which helps in identifying genetic relationships and similarity among individuals (Maguire and Woodward, 2008). Kinship analysis revealed the sufficient amount of genetic diversity in the association panel.

### 4.3 Genome-wide association study and candidate gene analysis

GWAS has been successfully utilized in identifying the significant MTAs associated with drought tolerance at various developmental stages (Gill et al., 2022; Tanin et al., 2022; Halder et al., 2023). Most of these studies were conducted at reproductive stages. However, the number of studies identifying MTAs for seedling drought stress tolerance are scarce (Ayalew et al., 2017; Ma et al., 2020; Maulana et al., 2020, Maulana et al., 2021; Ahmed et al., 2022; Schierenbeck et al., 2023). Therefore, in the present study, we carried GWAS using 12,511 SNPs and identified 39 MTAs associated with seedling characteristics under controlled and drought stress. Seed germination is key for maintaining optimum plant population in field as well as to maintain the stable yield under drought stress. Previous studies reported multiple QTLs for GP on different chromosomes (Landjeva et al., 2010). Similarly, we identified five MTAs associated with GP under drought stress on chromosomes 1A, 3D (2), 4A, and 6B. The SL is responsible for above-ground biomass production, light capture, resource allocation, biomass partitioning, structural adaptation, and reproductive success under drought stress (Gudi et al., 2022a). Several studies reported the significant MTAs associated with SL under drought stress on chromosomes 1A, 1B, 2A, 2B, 2D, 3A, 3B, 4A, 4B, 5A, 5B, 5D, 6A, 6B, 7A, and 7B (Sukumaran et al., 2018; Maulana et al., 2020; Schierenbeck et al., 2023). Similarly, in the present study, we identified three MTAs (on chromosomes 1A, 1B, and 2B) associated with SL under drought stress. Roots plays a critical role in plant adaptation to drought stress tolerance by enhancing water absorption and nutrient uptake. Several MTAs associated with root architecture and root biomass traits were identified on various chromosomes including, 2D, 5A and 6A (Bai et al., 2013), 5B, 6B, 7A, and 7B (Schierenbeck et al., 2023), and 1B and 4A (Breseghello and Sorrells, 2007). We also identified three MTAs associated with RL under drought stress condition and these MTAs were located on 6A, 6B, and 7A chromosomes. Coleoptile is the first plant organ which comes out by penetrating the soil and it will decide the seedling emergence under water stress. Candidate genomic regions associated with coleoptile related traits were identified on almost all the chromosomes (viz., 1A, 1B, 2A, 2B, 2D, 3A, 3B, 3D, 4A, 4B, 4D, 5A, 5B, 5D, 6A, 6B, 7A, and 7B) (Ma et al., 2020). In this GWAS, we identified four MTAs (on the chromosomes 2A, 4A, 6B, and 6D) associated with CL under drought stress. Vigor is the ability of a plant to thrive under unfavorable environments and support the plant establishment via efficient resource utilization. Previous studies identified the several QTLs associated with SV on chromosomes 1B, 2D, 4D, 5D, 6B, 6D and 7A (Moore and Rebetzke, 2015), 1D, 4D, and 5D (Landjeva et al., 2010), and 6A (Spielmeyer et al., 2007). Similarly, we identified six MTAs on 1B, 3A, 4A (2), 4B, and 6B chromosomes.

Multiple studies have reported that most of the identified MTAs are false positives or minor QTLs with minimum effect on phenotype (Gill et al., 2022; Gudi et al., 2022b). These MTAs not only weakens the effectiveness of marker-assisted selection (MAS) but also affect the trait introgression. Therefore, it is necessary to apply the more stringent criteria to eliminate the false positive QTL with minor effect on the phenotype without losing the major QTLs. In the present study, we applied such criteria and identified 12 MTAs as significant MTAs as they explained >10% phenotypic variance and had the LOD scores of >5. These MTAs were further utilized for extracting CG models.

Linkage disequilibrium (LD) is the non-random association of alleles at different genetic loci. It occurs when certain alleles at different loci are found together more frequently than would be expected by chance. LD is influenced by genetic distance, mode of pollination, recombination rate, and population history. Usually outbreeding species such as maize, sunflower, Alfalfa, etc. have the smaller LD block size (viz., 100-1500 Kb) in comparison to self-pollinated crops (up to 20 cM) such as wheat and rice (Vos et al., 2017). Several studies reported that the LD decay in wheat is greatly influenced by the type of populations used for GWAS and is varied across the different sub-genomes (Lin et al., 2019; Rathan et al., 2022). For instance, Devate et al. (2022) reported that the “sub-genome B” had the slower LD decay (viz., 5.64 Mb), whereas the “sub-genome A” had the faster LD decay (viz., 3.63 Mb) (Devate et al., 2022). However, Rathan et al. (2022) reported the slower decay in the “sub-genome B” (viz., 11.88 Mb) and the faster decay in the “sub-genome A” (viz., 3.63 Mb). In the present study we observed the similar trend as that observed by Rathan and co-authors (2022) with slower LD decays in “sub-genome B” (viz., largest LD block size of 11.88 Mb) and faster decay in the “sub-genome A” (viz., smallest LD block size of 5.91 Mb). The variations in LD decay among the three sub-genomes imply that each of these genomes and their diploid ancestors may originated independently and experienced distinct selection pressures during domestication. Furthermore, a significant difference in LD decay at the chromosome level was observed, with chromosome 7D exhibiting larger LD block (1.12 Mb), while chromosome 1D displaying smaller LD block (35.07 Mb). This indicates a differential rate of recombination among the chromosomes.

High-confidence MTAs identified through GWAS serve as potential targets for extracting CGs associated with the trait of interest. Several studies utilized GWAS-identified MTAs to extract the potential CGs associated with: (i) agronomic traits including days to anthesis, days to maturity, tiller number, spike length, spikelet number, grain number per spike, grain weight, and grain yield (Gill et al., 2022; Gudi et al., 2024); (ii) physiological traits such as chlorophyll fluorescence, chlorophyll content, vegetation index, gas exchange, and stomatal conductance (Hamdani et al., 2019; Gudi et al., 2023); (iii) stress tolerance such as drought, heat, salinity, etc (Tanin et al., 2022, Tanin et al., 2023; Tian et al., 2023); (iv) biochemical compounds such as proline, abscisic acid, and hydrogen peroxides (H_2_O_2_) (Verslues et al., 2014; Kamruzzaman et al., 2022); and (v) quality traits including grain protein content, sedimentation volume, kernel hardiness, solvent retention capacity, Fe content, and Zn content (Gudi et al., 2022b; Halladakeri et al., 2023). Similarly, in the present study we used 13 high-confidence MTAs explaining >10% phenotypic variance and having the LOD scores >5 to extract 216 CGs models. Of the 216 CGs, 83 gene models were found to be functionally associated with drought stress tolerance and were considered as the potential CGs. These genes were responsible for encoding following proteins: WD40 repeat domain, Myb/SANT-like domain, WSD1-like domain, BTB/POZ domain, protein kinase domain, cytochrome P450, leucine-rich repeat domain superfamily, BURP domain, calmodulin-binding protein60, ubiquitin-like domain, etc.

WD40 genes have been extensively studied for their regulatory role in a wide range of biological processes, such as grain yield and adaptation to various abiotic stresses, including drought. In wheat, WD40 gene located on chromosome 4B interacts with the canonical catalases to scavenge reactive oxygen species (ROS) and thereby provide high-level of drought stress tolerance (Tian et al., 2023). The Myb/SANT-like domains are responsible for providing drought stress tolerance by regulating the development of epidermis, stomatal cells, and trichomes as well as by regulating the auxin-salicylic acid cross-talk under the stress (Tiwari et al., 2020). Under drought stress, the presence of WSD1-like domains plays a vital role in protecting leaf tissues from dehydration. These domains facilitate the synthesis of cuticular wax, which acts as a barrier to prevent excessive water loss, thus safeguarding the leaf tissues from dehydration (Abdullah et al., 2021). The BTB/POZ domains upregulate the brassinosteroids signaling pathway and promote proline biosynthesis under drought stress, thereby mitigates the negative impact of ROS (Zhou et al., 2020). Protein kinases belong to the multi-gene family proteins responsible for tissue specific expression under stress conditions such as wounding, drought, heat, and salt stresses. Overexpression of protein kinase genes enhance the drought stress tolerance by preventing lipid peroxidation of cell membranes (Campo et al., 2014). BURP domains represent a plant-specific gene familiesthat play a crucial role in various biological processes, including drought stress tolerance. Proteins belonging to the BURP family, such as AtRD22 and AtUSPL1, have been identified as important contributors to drought tolerance in plants. These proteins are upregulated in response to drought stress, thereby confers drought stress tolerance (Harshavardhan et al., 2014). Although numerous genes associated with drought stress tolerance have been identified, their exact mechanisms of action remain unknown. Therefore, it is necessary to functionally validate these genes, which helps in understanding the metabolic processes and the intricate mechanisms involved in drought stress tolerance.

## 5 Conclusion

In the present investigation we identified the significant effects of genotypes and the drought stress on various seedling characteristics. Population structure, principal component, and kinship analysis revealed the presence of huge genetic variation in the association panel, which helps to identify 39 MTAs for different seedling traits under control and drought stress condition. The CGs analysis from the high-confidence MTAs identified 83 potential CGs associated with drought stress tolerance. The MTAs and CGs identified in this study may facilitate marker-assisted breeding to improve wheat varieties with enhanced drought stress tolerance. This can be done only when the identified MTAs and potential CGs are subjected to fine mapping and *in-silico* validation using reverse genetic or transgenic or genome editing approaches.

## Data availability statement

The data presented in the study are deposited in the DRYAD repository with the link https://datadryad.org/stash/share/utOBHq3aiZJDNRvIXVr-S8FvhlLfSa4m5_HhOAJh78Y.

## Author contributions

SG: Conceptualization, Data curation, Formal analysis, Methodology, Software, Validation, Visualization, Writing – original draft, Writing – review & editing. PH: Formal analysis, Software, Writing – original draft. GS: Data curation, Formal analysis, Writing – review & editing. PK: Data curation, Writing – review & editing. SS: Formal analysis, Writing – review & editing. KA: Formal analysis, Funding acquisition, Writing – review & editing. DM: Formal analysis, Funding acquisition, Writing – review & editing. AS: Conceptualization, Funding acquisition, Investigation, Project administration, Resources, Supervision, Writing – review & editing.

## Supplementary material

Supplementary Figure 1 Principal component analysis (PCA) for seedling traits under control and drought stress conditions: **(A)** scree plot under control condition; **(B)** PCA biplot under control condition; **(C)** scree plot under drought stress condition; and **(D)** PCA biplot under drought stress condition.

Supplementary Figure 2 Manhattan showing the quantitative trait loci (QTL) for germination percentage (GP) under control **(A)** and drought stress **(B)** conditions.

Supplementary Figure 3 Manhattan showing the quantitative trait loci (QTL) for shoot length (SL) under control **(A)** and drought stress **(B)** conditions.

Supplementary Figure 4 Manhattan showing the quantitative trait loci (QTL) for root length (RL) under control **(A)** and drought stress **(B)** conditions.

Supplementary Figure 5 Manhattan showing the quantitative trait loci (QTL)for coleoptile length (CL) under control **(A)** and drought stress **(B)** conditions.

Supplementary Figure 6 Manhattan and QQ-plots showing the quantitative trait loci (QTL) for seedling vigour (SV) control **(A)** and drought stress **(B)** conditions.

Supplementary Figure 7 Analysis of linkage disequilibrium (LD) at the chromosome level. The LD decay block size (in bp) is depicted in green color.
