# Supplementary figures and images for: Deciphering the genetic landscape of seedling drought stress tolerance in wheat (Triticum aestivum L.) through genome-wide association studies
Source: Front Plant Sci. 2024 Mar 4;15:1351075. doi: 10.3389/fpls.2024.1351075 (PMC10952099; doi:10.3389/fpls.2024.1351075)

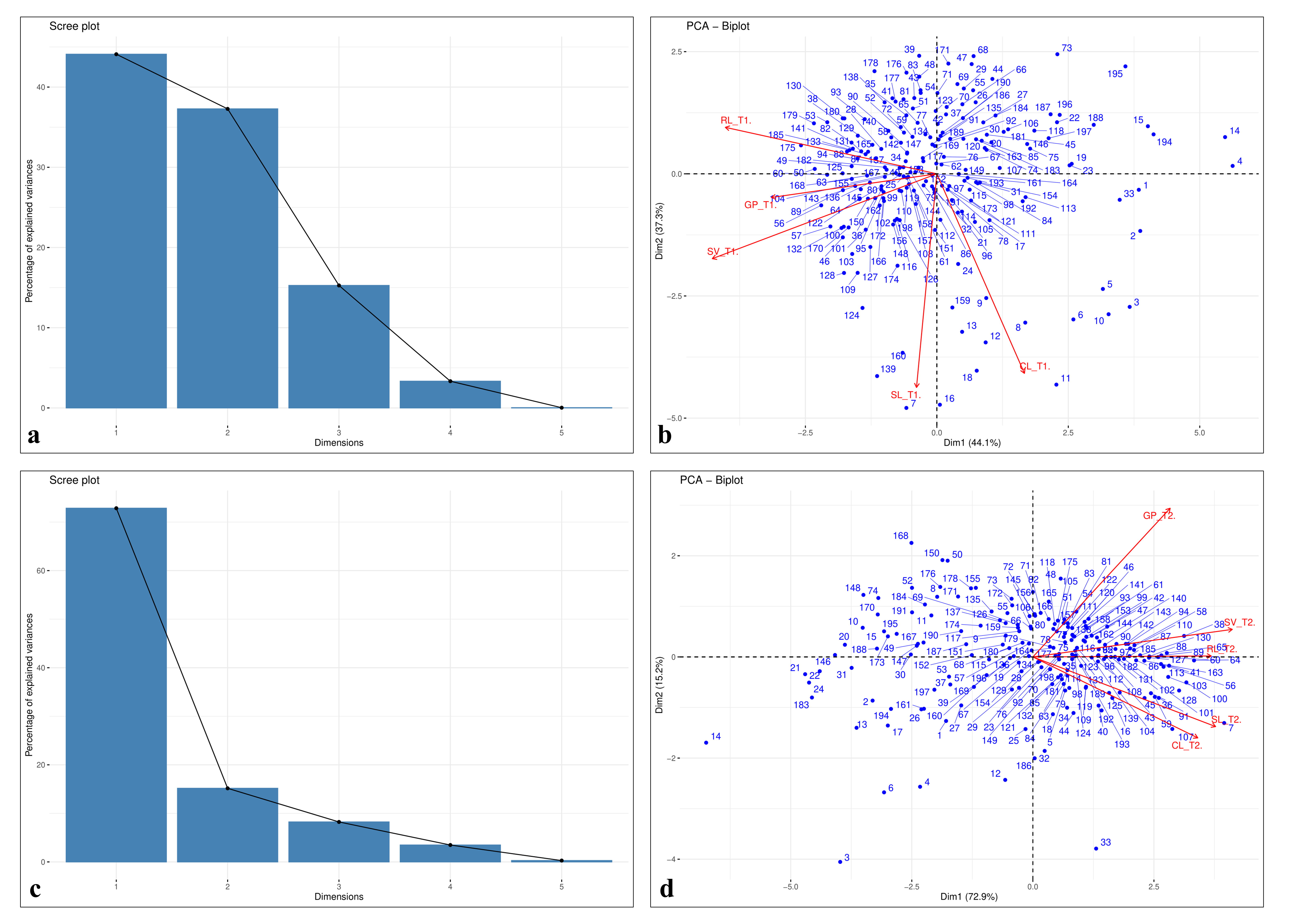

Supplement: Supplementary Figure 1 — Principal component analysis (PCA) for seedling traits under control and drought stress conditions: (A) scree plot under control condition; (B) PCA biplot under control condition; (C) scree plot under drought stress condition; and (D) PCA biplot under drought stress condition. [file Image_1.tif]

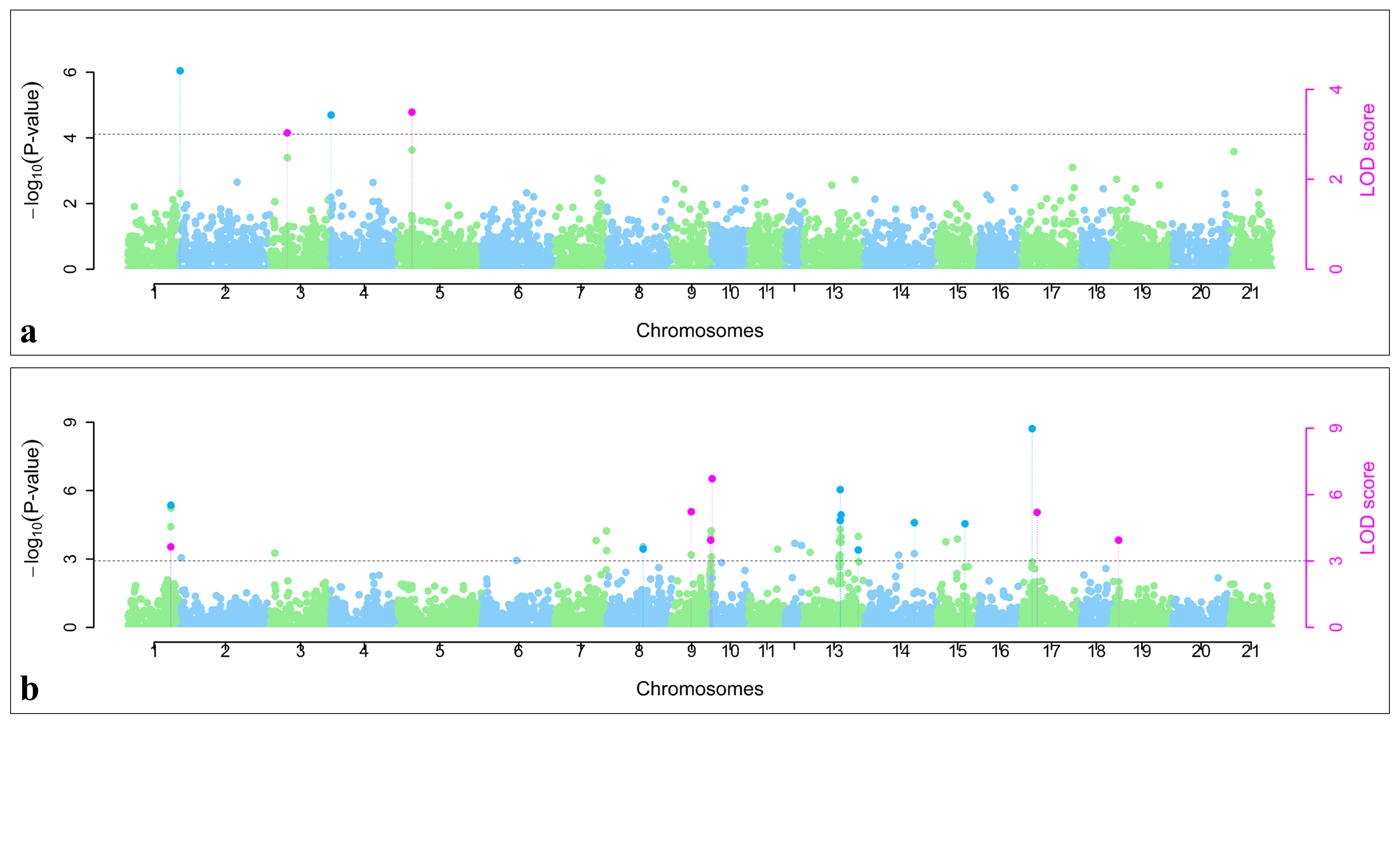

Supplement: Supplementary Figure 2 — Manhattan showing the quantitative trait loci (QTL) for germination percentage (GP) under control (A) and drought stress (B) conditions. [file Image_2.tif]

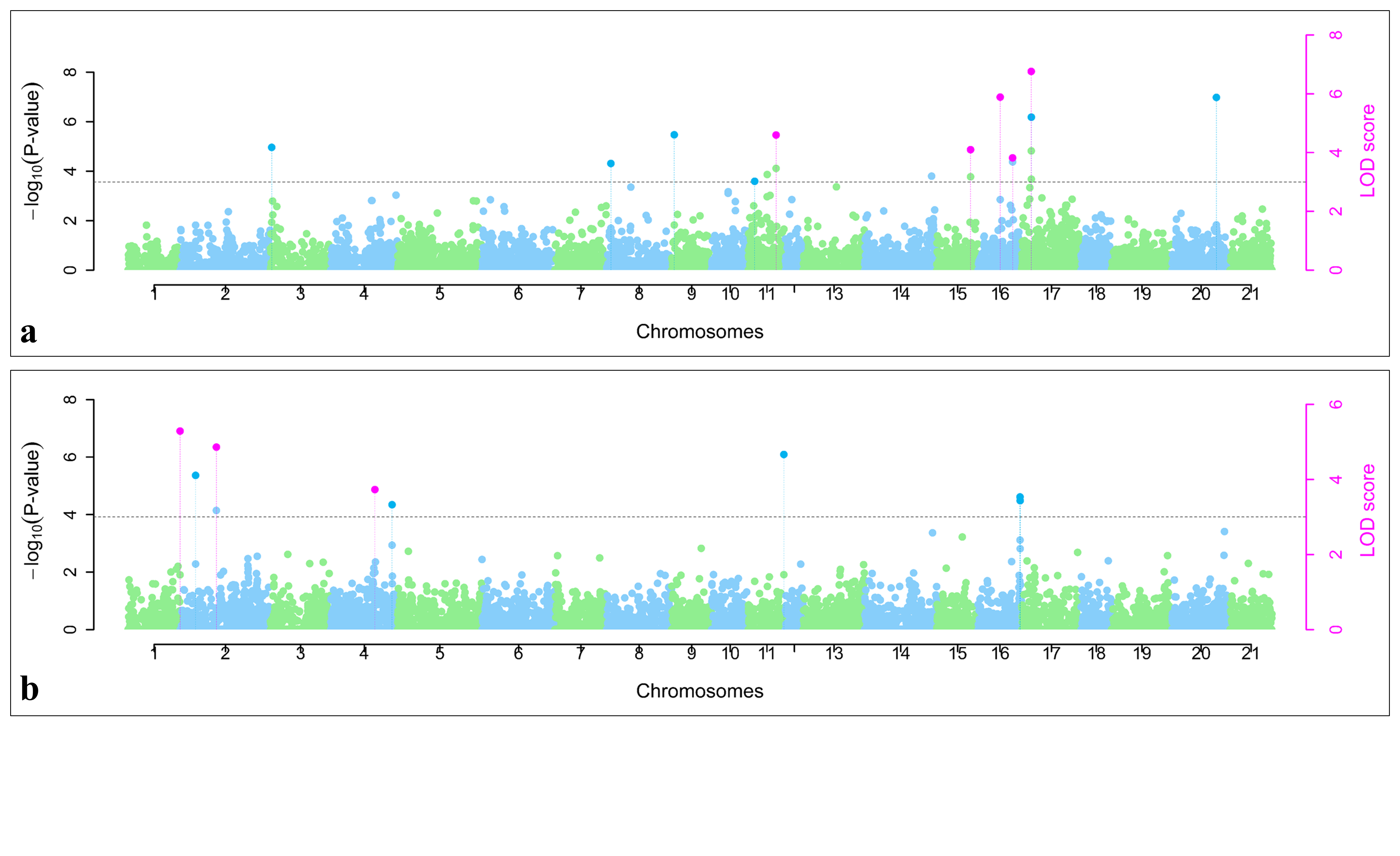

Supplement: Supplementary Figure 3 — Manhattan showing the quantitative trait loci (QTL) for shoot length (SL) under control (A) and drought stress (B) conditions. [file Image_3.tif]

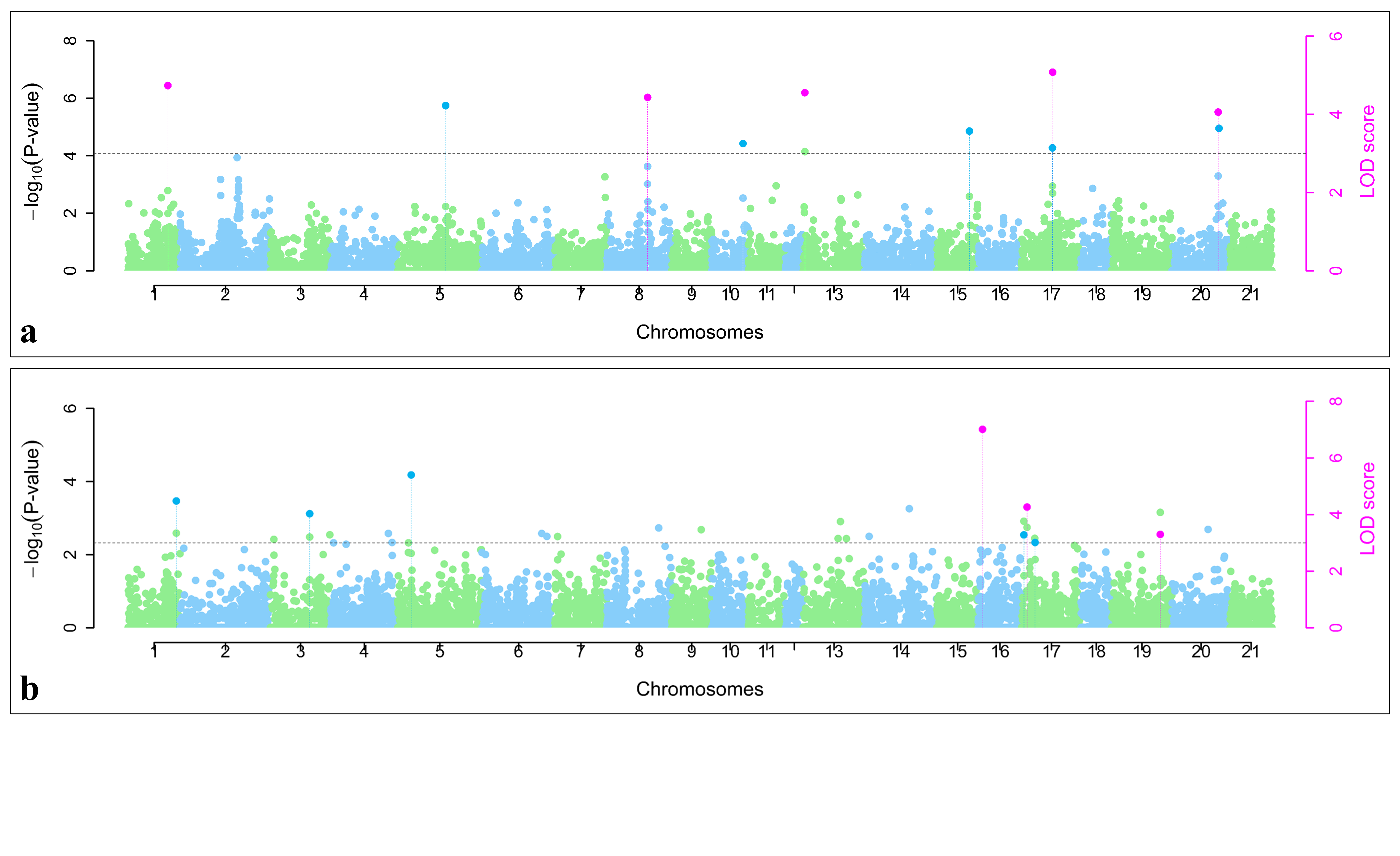

Supplement: Supplementary Figure 4 — Manhattan showing the quantitative trait loci (QTL) for root length (RL) under control (A) and drought stress (B) conditions. [file Image_4.tif]

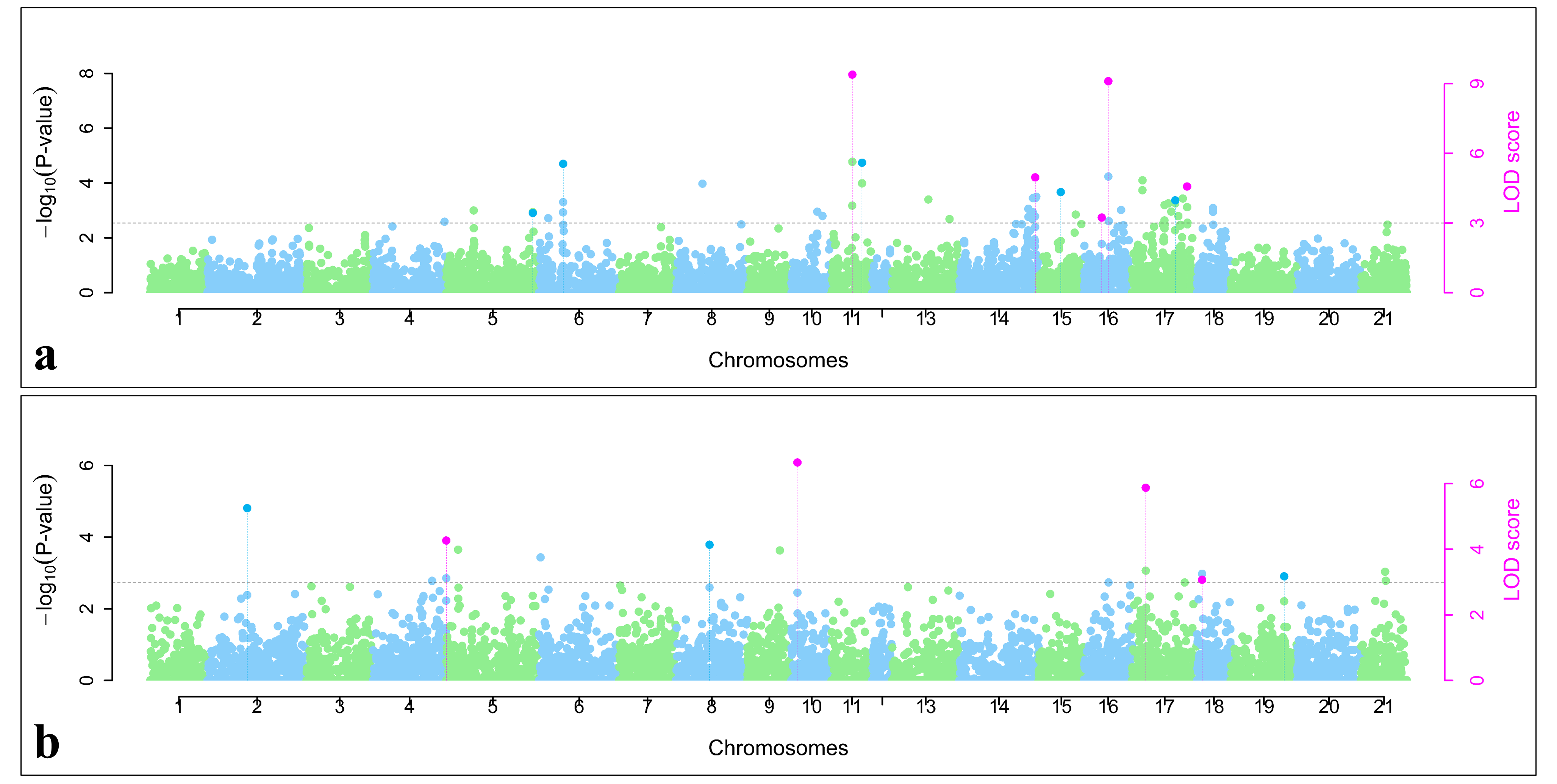

Supplement: Supplementary Figure 5 — Manhattan showing the quantitative trait loci (QTL)for coleoptile length (CL) under control (A) and drought stress (B) conditions. [file Image_5.tif]

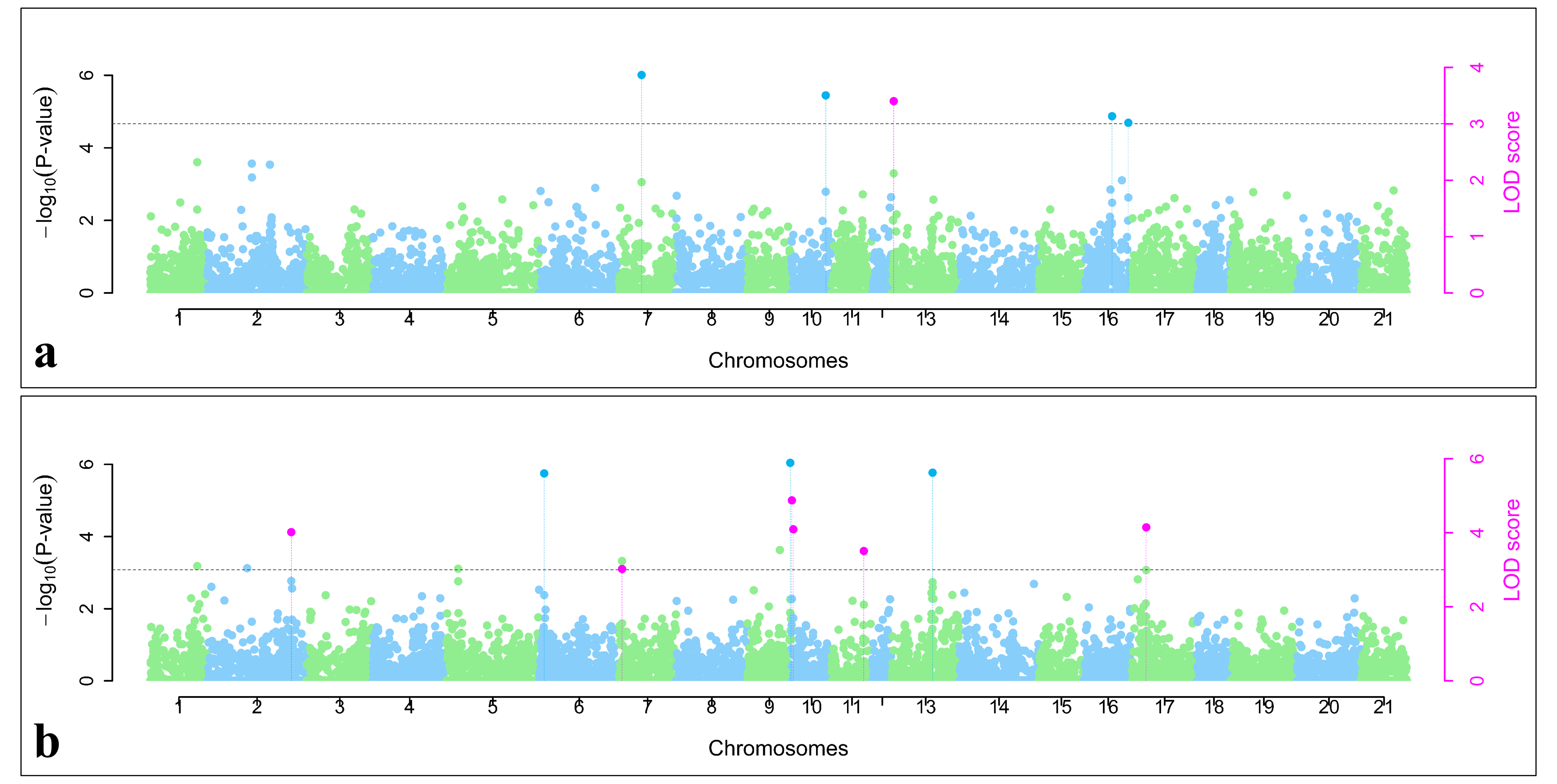

Supplement: Supplementary Figure 6 — Manhattan and QQ-plots showing the quantitative trait loci (QTL) for seedling vigour (SV) control (A) and drought stress (B) conditions. [file Image_6.tif]

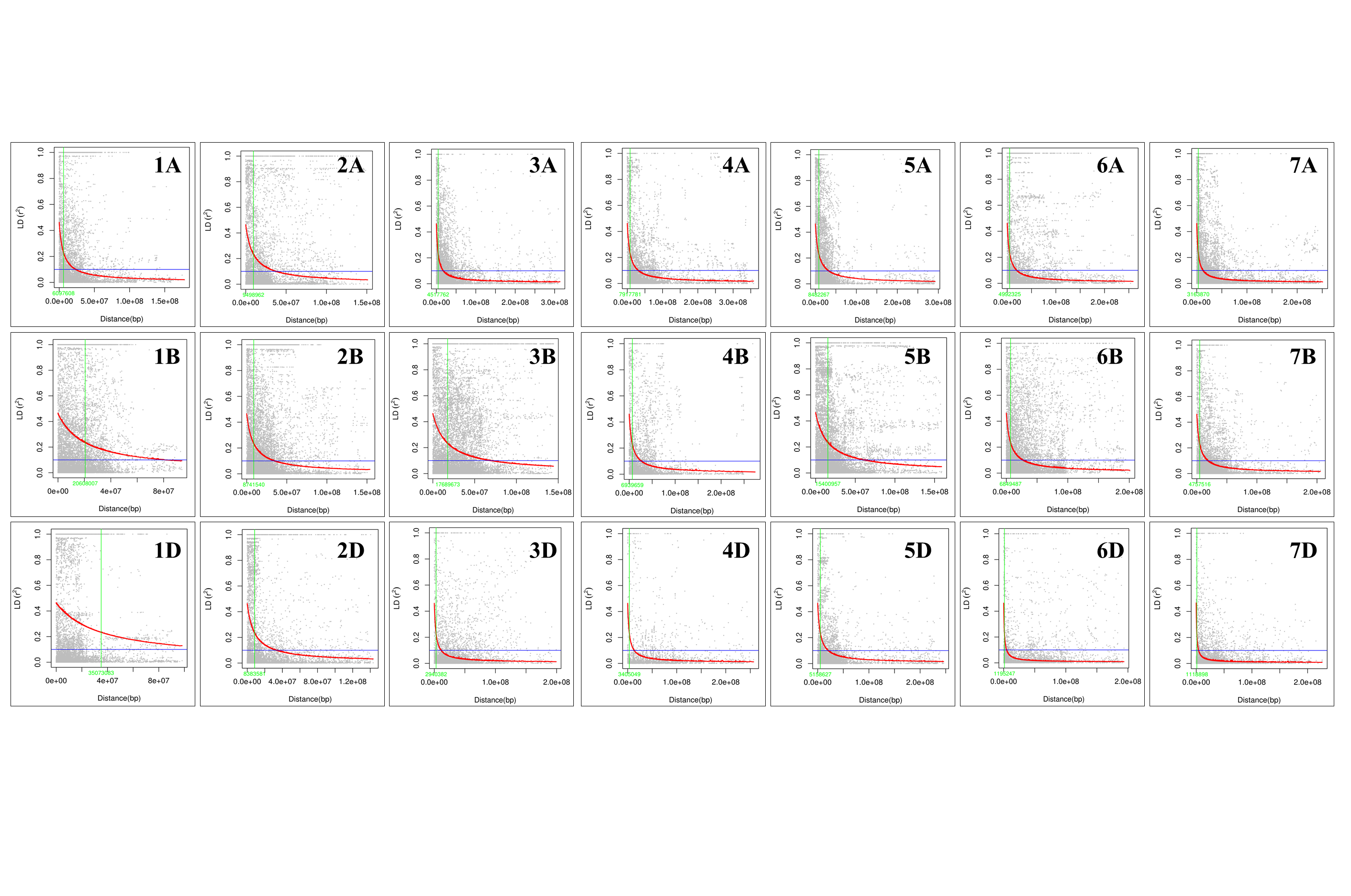

Supplement: Supplementary Figure 7 — Analysis of linkage disequilibrium (LD) at the chromosome level. The LD decay block size (in bp) is depicted in green color. [file Image_7.tif]
